# Female breast cancer incidence predisposing risk factors identification using nationwide big data: a matched nested case-control study in Taiwan

**DOI:** 10.1186/s12885-022-09913-6

**Published:** 2022-08-04

**Authors:** Ping-Hung Liu, James Cheng-Chung Wei, Yu-Hsun Wang, Ming-Hsin Yeh

**Affiliations:** 1 Division of General Surgery, Department of Surgery, Kaohsiung Armed Forces General Hospital, Kaohsiung, 81342 Taiwan; 2 Department of Breast and Thyroid Surgery, Chung Shan Medical University Hospital, Taichung, 404332 Taiwan; 3 Department of Allergy, Immunology and Rheumatology, Chung Shan Medical University Hospital, Taichung, 404332 Taiwan; 4 Department of Medical Research, Chung Shan Medical University Hospital, Taichung, 404332 Taiwan

**Keywords:** Breast cancer, Incidence risk, Predisposing factors, Multiple cancers, Heredity, Big data, Matched nested case-control study

## Abstract

**Background:**

Breast cancer is an umbrella term referring to a group of biologically and molecularly heterogeneous diseases originating from the breast. Globally, incidences of breast cancer has been increasing dramatically over the past decades. Analyses of multiple clinical “big data” can aid us in clarifying the means of preventing the disease. In addition, predisposing risk factors will be the most important issues if we can confirm their relevance. This study aims to provide an overview of the predisposing factors that contribute to a higher possibility of developing breast cancer and emphasize the signs that we ought to pay more attention to.

**Methods:**

This is a matched nested case-control study. The cohort focused on identifying the eligible risk factors in breast cancer development by data screening (2000-2013) from the Taiwan National Health Insurance Research Database (NHIRD) under approved protocol. A total of 486,069 females were enrolled from a nationwide sampled database, and 3281 females was elligible as breast cancer cohort, 478,574 females who had never diagnosed with breast cancer from 2000 to 2013 were eligible as non-breast cancer controls, and matched to breast cancer cases according to age using a 1:6 ratio.

**Results:**

We analyzed 3281 breast cancer cases and 19,686 non-breast cancer controls after an age-matched procedure. The significant predisposing factors associated with breast cancer development including obesity, hyperlipidemia, thyroid cancer and liver cancer. As for patients under the age of 55, gastric cancer does seem to have an impact on the development of breast cancer; compared with their counterparts over the age of 55, endometrial cancer appears to exhibit an evocative effect.

**Conclusions:**

In this nationwide matched nested case-control study, we identified obesity, hyperlipidemia, previous cancers of the thyroid, stomach and liver as risk factors associated with breast cancer. However, the retrospective nature and limited case numbers of certain cancers still difficult to provide robust evidence. Further prospective studies are necessitated to corroborate this finding in order to nip the disease in the bud.

**Trial registration:**

The studies involving human participants were reviewed and approved by the China Medical University Hospital [CMUH104-REC2-115(AR-4)].

**Supplementary Information:**

The online version contains supplementary material available at 10.1186/s12885-022-09913-6.

## Introduction

Over 2.1 million female (15% of all female with cancer) are diagnosed with breast cancer every year throughout the world [1]. Increased incidence of this cancer and its impact have turned it into a major problem. There are numerous risk factors such as sex, aging, estrogen, family history, gene mutations and unhealthy lifestyle, which can increase the possibility of developing breast cancer [2].

Causes of cancer can roughly be placed into two camps: factors we can control, and others beyond our control. The latter includes things like random changes to our genes as we get older, or those that are hereditary. By their nature, there is not much we can do about such risks. However, the many causes we do have some control over deserve our attention. Thus, identifying the root cause of the disease is a crucial issue. If we can act in advance, we can avoid harmful physical, psychological, and economic losses.

Workers must sharpen their tools first. Since we are living in the era of “Big Data”, the utilization of this brilliant implement allows us to integrate various sources of clinical, physiological and pathological information into consensus. Big data can give us a quick and correct analysis, unlike the shortcomings of being difficult to collect and easily missing in the past. More specifically, big data also can be helpful in developing and reshaping disease prevention strategies [3]. The purpose of this study is to provide an overview of the predisposing factors that contribute to a higher possibility of developing breast cancer and emphasize the signs that we ought to pay more attention to.

## Methods

### Data source

All data were retrospectively collected from Taiwan’s National Health Insurance Research Database (NHIRD), one of the largest administrative health care databases around the world and has been used widely in academic studies. We enrolled almost 99% of a population of 23 million beneficiaries in Taiwan. The database includes all insurance claims data, including outpatient visits, emergency admission, and hospitalization. Based on our study criteria, 1 million subjects were sampled from the 23 million beneficiaries and their data from 1999 to 2013 were collected. The sampled database was de-identification and the study was approved by the Institutional Review Board of Chung Shan Medical University Hospital [CMUH104-REC2-115(AR-4)]. Written informed consent for participation was waived for this study in accordance with the national legislation and the institutional requirements.

### Study cohort

**Fig. 1:**
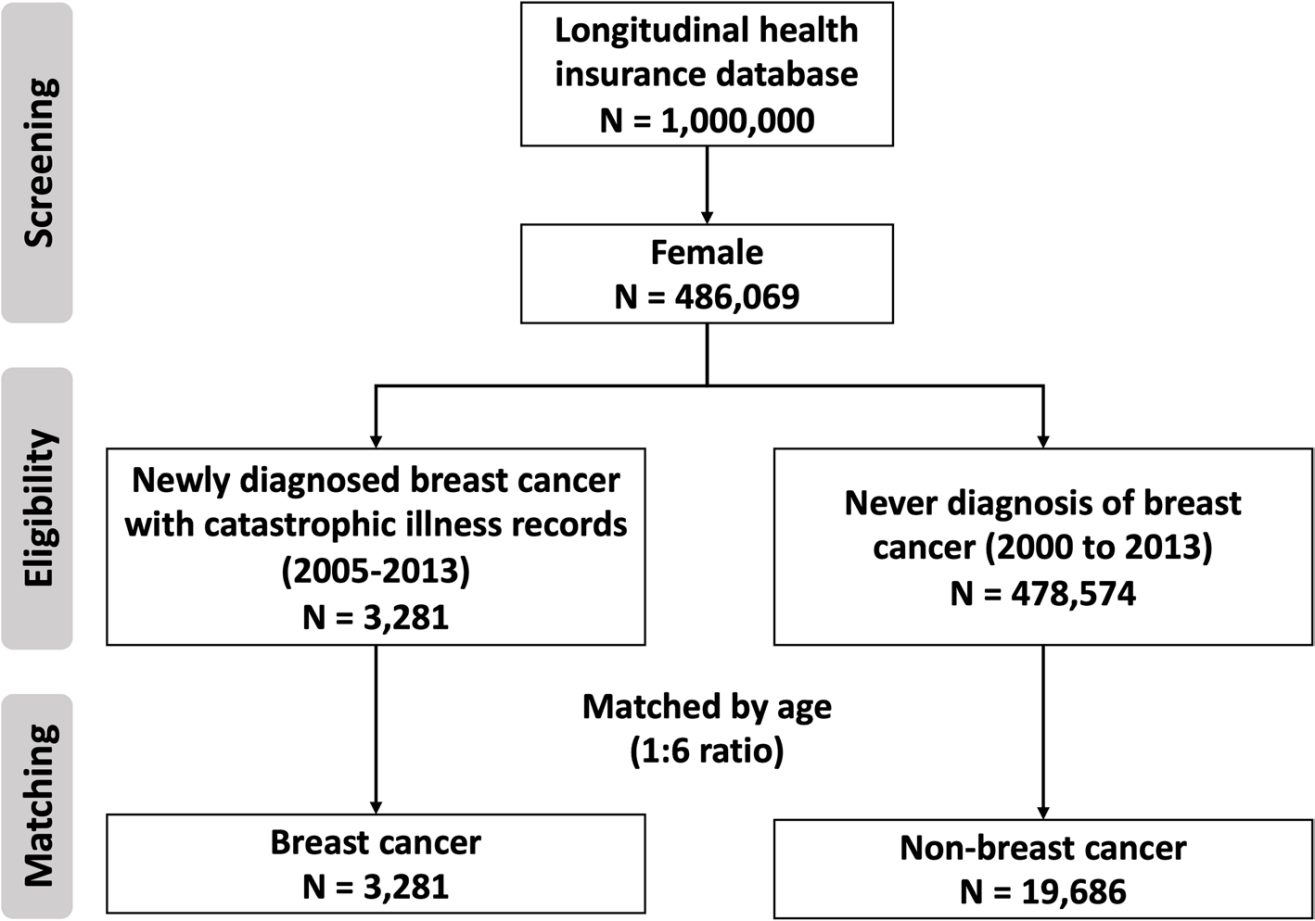
The study flowchart of matched nested case-control cohort based on nationwide sampled database

Since we want to conduct the comparative approach to the risk of breast cancer development in the fully enumerated cohort, we adopted the nested case-control study design to clarify their correlation. In order to verify the risk of pre-cancer, we set a backtracking period of five-years from the time of breast cancer diagnosis, and identified the diseases that were treated and meet the definition of ICD-9 code and identified at least three outpatient visits or one hospitalization. Of the 1 million samples, 486,069 females were enrolled, and 3281 females who was newly diagnosed with breast cancer (ICD-9-CM code 174) within 5 years after first observed date between 1 January 2005 and 31 December 2013, and obtained registry for catastrophic illness was eligible in breast cancer cohort. The first breast cancer diagnosis date was used as the index date for breast cancer cases. 478,574 females who had never diagnosed with breast cancer from 2000 to 2013 were eligible as non-breast cancer controls, and matched to breast cancer cases according to age using a 1:6 ratio. An index dates was set at the fifth year after the initial date of observation for non-breast cancer cohort. Hence, 19,686 matched controls were eligible in later analysis. The study flowchart was summarized in Fig.1.

### Variables

The baseline characteristics were age, gender, hypertension (ICD-9-CM codes 401-405), hyperlipidemia (ICD-9-CM codes 272.0-272.4), chronic liver disease (ICD-9-CM code 571), chronic kidney disease (ICD-9-CM code 585), diabetes (ICD-9-CM code 250), chronic obstructive pulmonary disease (ICD-9-CM codes 491, 492, 496), autoimmune disease (ICD-9-CM codes 710.0, 714.0, 720.0), cardiovascular disease (ICD-9-CM codes 410-414), stroke (ICD-9-CM codes 430-438), endometriosis (ICD-9-CM code 617), and obesity (ICD-9-CM code 278). Furthermore, we also obtained data of patients with cancers that may be related, such as colorectal cancer (ICD-9-CM codes 153, 154), lung cancer (ICD-9-CM code 162), thyroid cancer (ICD-9-CM code 193), liver cancer (ICD-9-CM code 155), cancer of the corpus uteri (ICD-9-CM code 182), ovary cancer (ICD-9-CM code 183), cervical cancer (ICD-9-CM code 180), skin cancer (ICD-9-CM codes 172, 173), and stomach cancer (ICD-9-CM code 151). The ICD-9-CM code for above mentioned disease were summarized in Supplementary Table S1. Thence, current study aimed at determining whether there is a positive correlation between suffering from certain diseases or cancers and the increased risk of breast cancer development.

### Statistical analysis

The chi-squared test or Student’s t-test was used to compare the demographic characteristics of breast cancer cases and non-breast cancer controls as appropriate. Conditional logistic regression was used to estimate the risk of breast cancer development in different cancer types, the odds ratio (ORs) and confidence interval (CI) were computed. In addition, since breast cancer is related to hormonal regulation, we added the average age of menopause at 55 years of age as a boundary to further clarify whether the age factor can be found to be related to the development of breast cancer from other cancer survivors. All *P* values were two-sided and *P* < 0.05 was considered statistical significance. All statistics was performed using SPSS version 18.0 (SPSS Inc., Chicago, IL, USA).

## Results

### Patient characteristics

In this case-control study, 486,069 females were included. Of these, 3281 individuals were afflicted with breast cancer, the rest were those who were not, but still had other chronic diseases or other types of cancer. The risk of developing breast cancer was elevated among the disease of hyperlipidemia and obesity (Table 1). For those who were already victims of cancer, there were several types of cancers that resulted in higher susceptibility to breast cancer later on, such thyroid cancer, and liver cancer (Table 2). After further subgroup correction when adjusted by age, we found that stomach cancer patients who were younger than the age of 55 years had a higher chance of getting breast cancer. In addition, endometrial cancer survivors who aged over 55 years were more likely to have breast cancer (Table 3).

**Table 1:** Demographic characteristics of breast cancer cases and non-breast cancer controls

Characteristics	Breast cancer (*N =* 3281)		Non-breast cancer (*N =* 19,686)		*P*
	*n*	%	*n*	%	
Age group					1.000
20-29	25	0.8	150	0.8	
30-39	311	9.5	1866	9.5	
40-49	974	29.7	5844	29.7	
50-59	1052	32.1	6312	32.1	
60-69	549	16.7	3294	16.7	
70-79	263	8.0	1578	8.0	
≥ 80	107	3.3	642	3.3	
Age, years (mean ± SD)	54.2 ± 12		54.2 ± 12		1.000
Hypertension	902	27.5	5164	26.2	0.130
Hyperlipidemia	609	18.6	3216	16.3	0.002
Chronic liver disease	307	9.4	1640	8.3	0.051
Chronic kidney disease	37	1.1	238	1.2	0.692
Diabetes	406	12.4	2299	11.7	0.252
COPD	153	4.7	886	4.5	0.678
Autoimmune diseases	58	1.8	321	1.6	0.568
Cardiovascular disease	285	8.7	1623	8.2	0.396
Stroke	152	4.6	1008	5.1	0.238
Endometriosis	77	2.3	472	2.4	0.860
Obesity	30	0.9	116	0.6	0.030
Colorectal Cancer	22	0.7	106	0.5	0.347
Lung cancer	11	0.3	49	0.2	0.370
Thyroid cancer	15	0.5	48	0.2	0.031
Liver cancer	18	0.5	59	0.3	0.022
Cancer of corpus uteri	6	0.2	20	0.1	0.200
Ovary cancer	2	0.1	19	0.1	0.533
Cervical cancer	18	0.5	90	0.5	0.478
Skin cancer	3	0.1	17	0.1	0.927
Stomach cancer	3	0.1	18	0.1	1.000

*COPD* Chronic obstructive pulmonary disease, *SD* Standard deviation

**Table 2:** Conditional logistic regression analysis for predisposing risk factors of breast cancer development

Predisposing risk factors	COR	95% CI	*P*	AOR^a^	95% CI	*P*
Colorectal Cancer	1.25	0.79-1.98	0.347	1.20	0.75-1.91	0.446
Lung cancer	1.35	0.70-2.59	0.372	1.31	0.68-2.52	0.421
Thyroid cancer	1.88	1.05-3.35	0.034	1.82	1.02-3.25	0.044
Liver cancer	1.84	1.08-3.13	0.024	1.78	1.04-3.06	0.037
Cancer of corpus uteri	1.80	0.72-4.48	0.207	1.93	0.77-4.83	0.162
Ovary cancer	0.63	0.15-2.72	0.533	0.57	0.13-2.50	0.459
Cervical cancer	1.20	0.72-2.00	0.479	1.21	0.72-2.01	0.471
Skin cancer	1.06	0.31-3.66	0.927	1.02	0.29-3.53	0.978
Stomach cancer	1.00	0.29-3.39	1.000	0.95	0.28-3.26	0.938

*COPD* Chronic obstructive pulmonary disease, *COR* Crude-odds ratio, *AOR* Adjusted-odds ratio, *CI* Confidence interval

^a^ Adjusted for colorectal cancer, lung cancer, thyroid cancer, liver cancer, cancer of corpus uteri, ovary cancer, cervical cancer, skin cancer, stomach cancer, hypertension, hyperlipidemia, chronic liver disease, chronic kidney disease, diabetes, COPD, autoimmune diseases, cardiovascular disease, stroke, endometriosis, and obesity

**Table 3:** Conditional logistic regression subgroup analysis for predisposing risk factors of breast cancer development

Predisposing risk factors	Age < 55			Age ≥ 55		
	AOR^a^	95% CI	*P*	AOR^b^	95% CI	*P*
Colorectal Cancer	1.25	0.47-3.32	0.648	1.18	0.69-2.00	0.548
Lung cancer	0.96	0.21-4.31	0.958	1.44	0.69-2.99	0.328
Thyroid cancer	2.57	1.22-5.40	0.013	1.23	0.47-3.25	0.670
Liver cancer	1.50	0.49-4.56	0.475	1.88	1.01-3.52	0.047
Cancer of corpus uteri	0.64	0.08-5.22	0.680	2.96	1.01-8.68	0.048
Ovary cancer	1.12	0.24-5.16	0.884	- ^c^	–	–
Cervical cancer	1.12	0.47-2.68	0.802	1.24	0.66-2.32	0.505
Skin cancer	-^c^	–	–	1.29	0.36-4.64	0.701
Stomach cancer	7.45	1.00-55.36	0.0498	0.34	0.04-2.58	0.296

*AOR* Adjusted-odds ratio, *CI* Confidence interval

^a^ Adjusted for colorectal cancer, lung cancer, thyroid cancer, liver cancer, cancer of corpus uteri, ovary cancer, cervical cancer, stomach cancer, hypertension, hypertension, hyperlipidemia, chronic liver disease, chronic kidney disease, diabetes, COPD, autoimmune diseases, cardiovascular disease, stroke, endometriosis, and obesity

^b^ Adjusted for colorectal cancer, lung cancer, thyroid cancer, liver cancer, cancer of corpus uteri, cervical cancer, skin cancer, stomach cancer, hypertension, hypertension, hyperlipidemia, chronic liver disease, chronic kidney disease, diabetes, COPD, autoimmune diseases, cardiovascular disease, stroke, endometriosis, and obesity

^c^ Omitted due to restricted sample size

### Chronic underlying disease

According to the study findings shown in Table 1, obesity has an obvious impact on the incidence of breast cancer (*P* = 0.030). Obesity is confirmed by the outpatient physician who obtained the ICD-9-CM codes as 278, which is generally a stricter definition. Although the number of breast cancer patients who meet obesity standards is 30, there are still obvious differences in risk compared with non-breast cancer patients. Hyperlipidemia also has a significant influence on the development of breast cancer (*P* = 0.002).

### Cancer survivors

According to the results of univariate analysis as shown in Table 2, the relationship between thyroid cancer survivor and breast cancer has been confirmed (*P* = 0.034). After adjusted by conditional logistic regression, the estimated OR of thyroid cancer was 1.82 (95% CI = 1.02-3.25), still remained significant higher risk in breast cancer development. In addition, liver cancer also showed significant higher risk in the future development of breast cancer, and the estimated OR was 1.78 (95% CI = 1.04-3.06, *P* = 0.022). As for the age adjusted subgroup analysis (Table 3), the result revealed that those aged over 55 years has exhibited a significant positive correlation when it comes to the development of breast cancer in endometrial cancer survivors with an OR of 2.96 (95% CI = 1.01-8.68, *P* = 0.048). For the opposing group consisting of those aged younger than 55, stomach cancer victims were reported to have a slightly significant higher risk of getting breast cancer with an OR of 7.45 (95% CI:1.00-55.36, *P* = 0.0498). Of notes, although a great odds of high incidence risk of breast cancer have been estimated for the stomach cancer victims, a conservative used of current results is suggested. To further clarify the potential effects varied by age, the conditional logistic regression subgroup analysis using an age cut-off value of 50 years old were summarized in Supplementary Table S2. However, the additional analysis results showed the included predisposing risk factors did not associated with the development risk of breast cancer in age < 50 years subgroup. In age ≥ 50 years subgroup, the liver cancer (OR = 2.32, 95% CI = 1.32-4.07, *P* = 0.003) was significant associated with the increasing risk of breast cancer development, which is consistent with the results found in age ≥ 55 years subgroup. In addition, although cancer of corpus uteri showed an potential increasing risk on breast cancer development, no statistical significance was estimated in age ≥ 55 years subgroup.

## Discussion

Obesity is a recognized risk factor for the development of breast cancer and its recurrence even when patients are treated appropriately [4]. Excessive estrogen production from expanded adipose tissue has been proposed as a possible trigger of breast cancer. Literary reviews have shown that overexposure to estrogen is associated with an increase in breast cancer risk with evidence of a dose-response relationship [5]. Another research also showed that estrogen and estrogen plus progestin can contribute significantly to the development of cancers, especially of the breast [6]. According to a meta-analysis by Liu et al., there is also a positive association showing that about a 5 kg/m2 rise in BMI resulted in about a 2% increase in breast cancer risk [7]. Therefore, obesity prevention is an indispensable issue when it comes to breast cancer treatment. In other words, obesity could be both a predictive and a prognostic factor of breast cancer, and is definitely worthy of more attention.

Hyperlipidemia, high blood cholesterol, is a common comorbidity to obesity [8]. There is also proof of the link between hyperlipidemia and the risk of breast cancer recurrence [9]. Hyperlipidemia and hyperglycemia were also comparatively more significant in patients with lymph node metastasis [10]. However, its impact as a risk factor for breast cancer is conflicting, and it is unclear whether total, LDL, or HDL cholesterol contributes to the disease. Kitahara et al. investigated the role of cholesterol and its associations with a number of cancers in a Korean registry of over 1 million patients and found that high cholesterol levels had a positive association with prostate, colon cancers in male and breast cancer in female [11]. By stark contrast, a large study in over 664,000 female utilizing Big Data from the UK Algorithm for Co-morbidity, Associations, Length of Stay and Mortality (ACALM) registry found that female above 40 years of age with high cholesterol levels were 45% less likely to develop breast cancer than those without high cholesterol [12]. The objective result in our study was that, hyperlipidemia indeed has a contributive impact on the incidence of breast cancer(*p*-value = 0.002). Nevertheless, further studies and literary reviews are needed to clarify the disparity in the findings in order to confirm the effect of cholesterol and its treatment on the etiology of breast cancer.

Cancer survivors can be affected by a number of health problems, but often their greatest concern is facing cancer again. Some cancer survivors may develop a new, unrelated cancer later on in life. We wanted to clarify which distinct cancers have connections to breast cancer, so we used the benefit of big data analyses to find the connection and discussed it further.

Thyroid cancer starts when healthy cells in the gland mutate and grow out of control. Breast and thyroid cancers are two malignancies with the highest incidence in female. These cancers often occur metachronously which means the two cancers have a successive relationship and occur more than 6 months later from the first episode based on Moertel’s definitions [13]. The risk of a subsequent second primary cancer, most often breast cancer, is increased for thyroid cancer survivors [14]. In the literature review, female with thyroid cancer have a 67% greater chance of developing breast cancer than the general population [15]. The incidence ratio of papillary thyroid cancer (PTC) in female and male was 3:1, and both the incidence and disease progression of PTC were potentially associated with the differential expression of sex hormones [16]. In addition, a bidirectional and causative association between breast cancer and thyroid cancer also have been reported previously [17], and female survivors of thyroid cancer are more likely to develop breast cancer including Asian population [18]. Previous study also indicates the female thyroid cancer with hormone receptor overexpression might increasing risk in developing metachronous breast tumors [19]. We suggest that these two malignancy share the same triggering factor, which is the sex hormone, estrogen. Besides, genes are also one of the common pathogenic factors between thyroid and breast cancer. A study on Swedish patients found that first-degree relatives of female diagnosed with breast cancer are at an increased risk of developing thyroid cancer [20]. Similar results were observed in a U.S. population [21]. There are two genetic factors that have been identified, PTEN and PARP. Phosphatase and tensin homolog (PTEN) is one of the most frequently mutated human tumor suppressor genes. Loss of PTEN activity, either at the protein or genomic level, has been related to many primary and metastatic malignancies including breast cancer [22]. Cowden Syndrome is a currently recognized genetic disorder, arising from mutations to the PTEN, which increases the risk of both breast and thyroid cancer [23]. PARP which stands for poly-ADP ribose polymerase, is also a famous target for breast cancer treatment, especially for those who have BRCA-1 and BRCA-2 mutations [24]. In addition, germline mutations in PARP4 were identified as a possible susceptible gene of primary thyroid and breast cancer [25]. The genetic connection in PARP family between breast cancer and thyroid cancer is still worth exploring and more research regarding the correlation is needed.

Based on our analysis, liver cancer survivors and secondary breast cancer had a significant correlation but has seldom been demonstrated before. The traceable reported case is a patient with hepatitis B who developed a tumor mass of the liver and was presented with right breast nodule at the same time [26]. From the literature review, cirrhosis has been proposed as a possible risk factor for male breast cancer [27]. Cirrhosis is also associated with increased levels of estrogen, which may be causally related to breast cancer [28]. Besides, people with cirrhosis have an increased risk of liver cancer and it is well-documented worldwide [29].

As for primary liver cancer (PLC), there is an unfavorable increasing trend observed in most developed countries, obesity and buildup of fat in the liver due to western dietary habits is the major reason of this tendency [30]. In other words, obesity and cirrhosis both attribute to incidences of liver cancer and breast cancer. In our subgroup analysis, a more significant relationship was evident in patients aged over 55 when compared to the young population (Table 3). This resembles the global incidence in PLC for the elderly and obese group, which may lead to a high prevalence of breast cancer followed by liver cancer. However, more evidence should be obtained to support this conclusion.

Stomach cancer is a genetically heterogeneous tumor with multifactorial etiologies, associated with environmental and genetic factors. We believe that if the two cancers share common genetic influence, there may be a positive correlation between the occurrence. BRCA1/2 and CDH1 (E-Cadherin) are genes that are evident in the literature review. First, females with a mutated *BRCA1/2* gene are five times more likely to develop breast cancer than someone without a mutation [31]. Stomach cancer represents a significant global cancer burden and *BRCA1/2* mutations have been reported to increase the lifetime risk of developing stomach cancer by as much as 6 folds among first-degree relatives of *BRCA1/2* mutation carriers [32]. Second, the cell surface glycoprotein E-cadherin (CDH1) is a key regulator of adhesive properties in epithelial cells. Somatic CDH1 mutations have been identified in approximately 50% of sporadic diffuse gastric tumors and lobular breast cancers but rarely occur in other tumors [33]. Other research also confirms that female with CDH1 mutations have a significant lifetime risk of breast cancer as well as diffuse gastric cancer. Apart from this, excess body weight increases a man’s risk of developing stomach cancer. Furthermore, obesity and gastroesophageal reflux disease have specifically been related to an increase in the risk of cardia gastric cancer [34]. If obesity contributes to the development of stomach cancer, it will have the same cumulative effect on breast cancer development as the individual ages. It is still unclear as to why the positive correlation was restricted in those under the age of 55, but the results from present investigations seem worthy of being noted.

Cancer of corpus uterine (endometrial cancer) begins in the layer of cells that from the lining of the uterus. The known risk factors are hormone related, such as early menarche or late menopause, nullipara, irregular menstrual periods and obesity. Exogenous hormones can increase the risk of developing endometrial cancer. A large retrospective meta-analysis in German and Swedish Cancer Registry discovered that elevated risks were present on second ovarian endometrial carcinoma and secondary kidney cancer for 3973 endometrial cancer survivors [35], nothing was mentioned regarding breast cancer. However, the trend of developing breast cancer and colon cancer after endometrial cancer can be seen in the statistics put forth by the American Cancer Society’s medical and editorial content team, but it is not well-discussed [36]. In our retrospective analysis, we also favored the increased risk of developing breast cancer by endometrial cancer survivors, especially among the group over 55 years of age. We hypothesize that this may be affected by the cumulative influence of hormone stimulation. A woman’s hormonal balance plays a crucial part in the development of most endometrial cancers. A shift in the balance of these hormones toward a higher estrogen level increases a woman’s risk for endometrial cancer [36]. After menopause, the ovaries stop making these hormones, but a small amount of estrogen is still made naturally in fat tissue. Estrogen from fat tissue has a bigger impact after menopause than it does before menopause. We can find that post-menopausal female (age > 55) have increasing chances when it comes to the development of breast cancer (Table 3). The female who are susceptible to endometrial cancer may be due to their sensitivity to estrogen, which means that long-term exposure to estrogen can also be the cause of breast cancer. However, we need a larger sample size study to confirm this association.

The enrolled samples reviewed in this study have three potential limitations. The first relates to the size of some comparison groups, especially for cancer survivors. Since we set strict conditions for the continued development of cancer, some cancers have only single digits, such as cancer of corpus uteri, ovary cancer, skin cancer and stomach cancer. These samples were too small to precisely detect risk factors with low prevalence number. The second limitation is the background population, they were all restricted to the same race and a single country. It cannot be completely regard as the tendency of most people. The third limitation relates to self-reported data which can introduce a bias. Take obesity for example, its association with hormone related cancers has been accepted, but obesity is not commonly diagnosed as a disease, unless interventional treatment, such as surgery, is required. Therefore, the input of this ICD-9-CM code may be indirectly underestimated. The same incident may easily occur on the input of some chronic diseases. Moreover, the analysis data of current study were only retrospectively abstracted from the outpatient visits, emergency admission, and hospitalization records registered in Taiwan’s NHIRD, the individual information including genetic factors and family history for study cohort, and tumor characteristics for cancer patients are not available. Despite this, this study still provided a nationwide evidence to identify predisposing risk factor associated with breast cancer development, and carefully discussed the potential association between the predisposing factors and breast cancer development according to the our study findings.

## Conclusion

To sum up, this article has presented a retrospective nationwide matched nested case-control study based on the basic concept of “Big Data” integration, and further revealed several predisposing factors associated with breast cancer incidence risk. Breast cancer prevention requires raising awareness among those possible predisposing risk factors. However, the retrospective nature and limited case numbers of certain cancers still difficult to provide robust evidence. Therefore, further prospective research and investigation need to be carried out in order to achieve greater consensus.

## Supplementary Information

**Additional file 1: Supplementary Table S1**. The ICD-9-CM code for all analyzed diseases.

**Additional file 2: Supplementary Table S2**. Conditional logistic regression subgroup analysis for predisposing risk factors of breast cancer development.

## Data Availability

The datasets analyzed during the current study are not publicly available due the regulation of institution but secondary data are available from the corresponding author on reasonable request.

## Abbreviations

NHIRD: National Health Insurance Research Database
ICD-9-CM: International Classification of Diseases, Ninth Revision, Clinical Modification
COPD: Chronic obstructive pulmonary disease
OR: Odds ratio
CI: Confidence interval
LDL: Low-Density Lipoprotein
HDL: High-Density Lipoprotein
ACALM: Algorithm for Co-morbidity, Associations, Length of Stay and Mortality
PTC: Papillary thyroid cancer
PTEN: Phosphatase and TENsin homolog
PARP: Poly ADP-Ribose Polymerase
BRCA1/2: BReast CAncer gene 1/2
PLC: Primary liver cancer
CDH1: E-Cadherin gene
