# Supplementary material for: Female breast cancer incidence predisposing risk factors identification using nationwide big data: a matched nested case-control study in Taiwan
Source: BMC Cancer. 2022 Aug 4;22:849. doi: 10.1186/s12885-022-09913-6 (PMC9351234; doi:10.1186/s12885-022-09913-6)
Supplement: Supplementary file 1 — Additional file 1: Supplementary Table S1. The ICD-9-CM code for all analyzed diseases. [file 12885_2022_9913_MOESM1_ESM.docx]

**Supplementary Table S1**. The ICD-9-CM code for all analyzed diseases.

| **Diseases** | **ICD-9-CM codes** |
| --- | --- |
| Breast cancer | 174 |
| Hypertension | 401-405 |
| Hyperlipidemia | 272.0-272.4 |
| Chronic liver disease | 571 |
| Chronic kidney disease | 585 |
| Diabetes | 250 |
| Chronic obstructive pulmonary disease (COPD) | 491, 492, 496 |
| Autoimmune diseases | 714.0, 720.0, 710.0 |
| Cardiovascular disease | 410-414 |
| Stroke | 430-438 |
| Endometriosis | 617 |
| Obesity | 278 |
| Colorectal Cancer | 153, 154 |
| Lung cancer | 162 |
| Thyroid cancer | 193 |
| Liver cancer | 155 |
| Cancer of corpus uteri | 182 |
| Ovary cancer | 183 |
| Cervical cancer | 180 |
| Skin cancer | 172, 173 |
| Stomach cancer | 151 |

ICD-9-CM: International Classification of Diseases, Ninth Revision, Clinical Modification.
