# Supplementary material for: Female breast cancer incidence predisposing risk factors identification using nationwide big data: a matched nested case-control study in Taiwan
Source: BMC Cancer. 2022 Aug 4;22:849. doi: 10.1186/s12885-022-09913-6 (PMC9351234; doi:10.1186/s12885-022-09913-6)
Supplement: Supplementary file 2 — Additional file 2: Supplementary Table S2. Conditional logistic regression subgroup analysis for predisposing risk factors of breast cancer development. [file 12885_2022_9913_MOESM2_ESM.docx]

**Supplementary Table S2.** Conditional logistic regression subgroup analysis for predisposing risk factors of breast cancer development.

| Predisposing risk factors | Age <50 | | |  | Age ≥50 | | |
| --- | --- | --- | --- | --- | --- | --- | --- |
|  | AOR^a^ | 95% CI | *P* |  | AOR^b^ | 95% C.I. | *P* |
| Colorectal Cancer | 0.39 | 0.05-2.94 | 0.360 |  | 1.33 | 0.82-2.15 | 0.248 |
| Lung cancer | 1.63 | 0.18-14.84 | 0.664 |  | 1.31 | 0.66-2.61 | 0.438 |
| Thyroid cancer | 1.74 | 0.70-4.35 | 0.236 |  | 1.97 | 0.92-4.20 | 0.080 |
| Liver cancer | -^c^ | - | - |  | 2.32 | 1.32-4.07 | 0.003 |
| Cancer of corpus uteri | 0.94 | 0.11-8.02 | 0.954 |  | 2.32 | 0.83-6.50 | 0.110 |
| Ovary cancer | 0.72 | 0.09-5.81 | 0.754 |  | 0.47 | 0.06-3.76 | 0.479 |
| Cervical cancer | 0.90 | 0.20-4.01 | 0.891 |  | 1.24 | 0.72-2.14 | 0.430 |
| Skin cancer | -^c^ | - | - |  | 1.12 | 0.32-3.96 | 0.862 |
| Stomach cancer | -^c^ | - | - |  | 0.30 | 0.04-2.28 | 0.245 |

AOR: adjusted-odds ratio; CI: confidence interval.

^a^ Adjusted for colorectal cancer, lung cancer, thyroid cancer, cancer of corpus uteri, ovary cancer, cervical cancer, hypertension, hypertension, hyperlipidemia, chronic liver disease, chronic kidney disease, diabetes, COPD, autoimmune diseases, cardiovascular disease, stroke, endometriosis, and obesity.

^b^ Adjusted for colorectal cancer, lung cancer, thyroid cancer, liver cancer, cancer of corpus uteri, ovary cancer, cervical cancer, skin cancer, stomach cancer, hypertension, hypertension, hyperlipidemia, chronic liver disease, chronic kidney disease, diabetes, COPD, autoimmune diseases, cardiovascular disease, stroke, endometriosis, and obesity.

^c^ Omitted due to restricted sample size.
